# Joint effect of smoking and *NQO1* C609T polymorphism on undifferentiated nasopharyngeal carcinoma risk in a North African population

**DOI:** 10.1002/mgg3.461

**Published:** 2018-08-29

**Authors:** Khalid Moumad, Wafa Khaali, Abdellatif Benider, Wided Ben Ayoub, Mokhtar Hamdi‐Cherif, Kada Boualga, Elham Hassen, El Khalil Ben Driss, Marilys Corbex, Meriem Khyatti

**Affiliations:** ^1^ Oncovirology Laboratory Institut Pasteur du Maroc Casablanca Morocco; ^2^ Departement of Biology, Faculty of Sciences Abdelmalek Essaadi University Tetouan Morocco; ^3^ Service de Radiothérapie Centre d'oncologie Ibn Rochd Casablanca Morocco; ^4^ Association Tunisienne de Lutte Contre le Cancer Tunis Tunisia; ^5^ Service d'épidémiologie CHU de Sétif Sétif Algeria; ^6^ Service de Radiothérapie Oncologique Centre Antic‐Cancer de Blida Blida Algeria; ^7^ Molecular Immuno‐Oncology Laboratory, Faculty of Medicine Monastir University Monastir Tunisia; ^8^ Who Regional Office for Europe Copenhagen Denmark

**Keywords:** nasopharyngeal carcinoma, North Africa, *NQO1* polymorphism, smoking

## Abstract

**Background:**

Nasopharyngeal carcinoma (NPC) has a higher incidence in North Africa than in most parts of the world. In addition to environmental factors such as Epstein–Barr virus infection and chemical carcinogen exposure, genetic susceptibility has been reported to play a key role in the development of NPC. NAD(P)H: quinone oxidoreductase 1 is a cytosolic enzyme that protects cells from oxidative damage. A C to T transition at position 609 in the *NQO1* gene (OMIM: 125860) has been shown to alter the enzymatic activity of the enzyme and has been associated with increased risk to several cancers. This study investigates for the first time the effect of this polymorphism on NPC susceptibility in a North African population.

**Methods:**

The *NQO1* C609T polymorphism was genotyped using PCR‐RFLP in 392 NPC cases and 365 controls from Morocco, Algeria, and Tunisia.

**Results:**

The allele frequencies and distributions of genotypes did not differ between cases and controls (*p* > 0.05). When stratifying according to smoking status, we observed two‐fold higher NPC risk in ever‐smokers carrying the CT or TT genotype. Multiple logistic regression analysis revealed that there was a significant interaction between T allele and smoking status (OR = 1.95, 95% CI = 1.20–3.19; interaction *p* = 0.007).

**Conclusion:**

In this North African population, the functional *NQO1* polymorphism was associated with a significantly higher risk of NPC among smokers and did not affect the risk among nonsmokers.

## 1 INTRODUCTION

Nasopharyngeal carcinoma (NPC) is a disease with distinct ethnic and geographic distribution. It is rare in most parts of the world but is frequent in Southern China and some parts of South‐East Asia, with an incidence rate of about 20 new cases per year per 100,000 inhabitants. Intermediate rates of NPC are observed, in natives of the Arctic region and North Africa as well as several populations in South‐East Asia (Busson, Keryer, Ooka, & Corbex, 2004; Chang & Adami, 2006).Throughout the world, NPC accounts for 84,000 new cases and 51,000 deaths annually (Ferlay et al., 2010). The etiology of NPC is complex and includes viral, genetic, and environmental factors. It is established that the Epstein–Barr virus (EBV) infection plays a major role in the pathogenesis of NPC in both endemic and nonendemic areas (Razak et al., 2010).

As for many cancers, genetic polymorphisms may determine individual's susceptibility to the development of NPC. The identification of single‐nucleotide polymorphisms (SNP) that affect gene function or expression and contribute to NPC susceptibility is important as it may help to predict individuals at risk. It also contributes to clarify pathophysiologic mechanisms relevant to NPC, its prevention and treatment (Hildesheim & Wang, 2012; Tse et al., 2009).

The NAD(P)H quinone oxidoreductase 1 (NQO1) is a cytosolic enzyme that catalyzes the 2‐electron reduction of quinones to hydroquinones. This can have both beneficial (detoxification) and harmful effects (activation of procarcinogens) for the cell. But overall, NQO1 is expected to protect the cell against cytotoxicity by reducing the concentration of free quinone available for single electron reduction. It can notably protect the cells from various types of carcinogens contained in tobacco smoke, by participating in their metabolic activation and detoxification (O'Brien, 1991).

There have been more than 93 SNPs identified in the *NQO1* gene (OMIM: 125,860). The more prominent one, in terms of both frequency and phenotypic consequences, is a C to T change at position 609 of the *NQO1* cDNA. This nucleotide substitution results in a proline to serine substitution at position 187 of the amino acid sequence of the protein and occurs at a frequency of 14%–20% in Caucasians, 22% in African American, and to 44% in Asians (Kelsey et al., 1997). Frequencies in the North African population are not known.

Association between tobacco consumption and risk of differentiated NPC (WHO Type I, differentiated squamous cell), which represent the majority of NPC in nonendemic region, is well established (Jia & Qin, 2012). Association with undifferentiated NPC (UCNT) is not as clear. Case–control studies from endemic areas (mainly from Asia), where the vast majority of NPCs are UCNTs, have produced conflicting results (Armstrong et al., 2000; Chen et al., 1990; Cheng et al., 1999; Friborg et al., 2007; Guo et al., 2009; Ning, Yu, Wang, & Henderson, 1990; Yu, Garabrant, Huang, & Henderson, 1990; Zou et al., 2000).

In North Africa, our study is the first to investigate the association between tobacco smoking and NPC. In the present sample, we found a significant association between cigarette smoking and differentiated NPC but not with UCNT, the major histological type in the sample. However a trend of increased risk of UCNT with increased dose of cigarette intake per day was apparent, the OR of ever‐smokers compared to nonsmokers was 1.3, but this did not reach statistical significance (Feng et al., 2009).

Previous studies of the association between the *NQO1* C609T polymorphism and human cancers other than NPC resulted in mixed findings. The C609T polymorphism has been associated with an increased risk of bladder cancer (Park, Zhao, Spitz, Grossman, & Wu, 2003), cervical cancer (Niwa et al., 2005), urothelial cancer (Wang, Lee, Tseng, Shen, & Chiou, 2008), colorectal cancer (van der Logt et al., 2006), and adult leukemias (Naoe et al., 2000). In contrast, no association was found in gastric cancer (Hamajima et al., 2002), head and neck cancers (Begleiter et al., 2005), adult gliomas (Peters et al., 2001), lymphomas (Soucek, Sarmanová, Kristensen, Apltauerová, & Gut, 2002) and conflicting results have been published for lung cancer (Kiyohara, Yoshimasu, Takayama, & Nakanishi, 2005), breast cancer (Menzel et al., 2004), and esophageal cancer (di Martino et al., 2007; Hamajima et al., 2002; Sarbia et al., 2003; Zhang, Li, et al., 2003; Zhang, Schulz, et al., 2003).

In this study, we studied the association of *NQO1* C609T polymorphism and risk of NPC among North Africans.

## 2 MATERIALS AND METHODS

### 2.1 Study population

Details of the studied populations are described elsewhere (Feng et al., 2007, 2009 ). The study was designed by the International Agency for Research on Cancer (IARC), the recruitment took place between 2002 and 2005. In brief, 636 NPC cases were recruited from five hospitals in Morocco, Algeria, and Tunisia, together with 615 controls frequency matched by center, age, sex, and childhood household type (urban/rural). Inclusion criteria requested that the four grandparents of each subject were of Moroccan, Algerian, or Tunisian origin. The IARC ethical committee approved the study protocol. For the study of *NQO1* polymorphism, only the samples with high amount of DNA left, and only cases with undifferentiated NPC were considered (the differentiated NPC case sample being too small) that is 392 cases and 365 controls, were used.

### 2.2 Genotyping

A polymerase chain reaction (PCR)‐restriction fragment length polymorphism‐based assay was used to determine the *NQO1* (rs1800566) genotype. PCR products were generated using 60 ng of genomic DNA. The forward primer 5′‐TCCTCAGAGTGGCATTCTGC‐3′ and reverse primer 5′‐TCTCCTCATCCTGTACCTCT‐3′ were used to amplify a 230‐bp oligonucleotide that included the *NQO1* C609T polymorphism (NM_000903.2:c.559C>T). This C/T transition creates a new HinfI restriction site that is used to distinguish the variant from the wild‐type alleles. The PCR was carried out using a 25 µl reaction with a final concentration of 0.2 mM dNTP, 2.0 mM MgCl2, 0.2 µM each primer, and 1 unit of Taq polymerase (Go Taq DNA Polymerase, Promega). After an initial denaturation at 95°C for 5 min, the cycling conditions were as follows: 35 cycles at 95°C for 30 s, 58°C for 30 s, and 72°C for 45 s, with one final extension at 72°C for 5 min. The PCR product was digested by Hinf1 enzyme. The variants (size of bands for each allele) were determined by a 3% agarose gel. Internal blinded quality control samples (20% of samples in duplicate) were 100% concordant.

### 2.3 Statistical analysis

The genotype frequencies in cases and controls were tested for Hardy–Weinberg equilibrium (HWE) using a Pearson goodness‐of‐fit test (https://ihg2.helmholtz-muenchen.de/cgi-bin/hw/hwa1.pl). Adjusted odds ratios (ORs) and their corresponding confidence intervals (CI) were estimated using multiple logistic regressions after inclusion of matching variables (center, age, sex, and childhood household type). Ever‐smokers were defined as individuals who have smoked at least 100 cigarettes in their lifetime, or as individuals who, at the time of the survey, smoked cigarettes. We estimated the possible interactions between *NQO1* C609T polymorphism and smoking on the risk of NPC using multiple logistic regression analysis. All statistical analyses were performed using SPSS statistical software (SPSS 17.0 Inc., Chicago); a *p* < 0.05 was considered statistically significant.

## 3 RESULTS AND DISCUSSION

The genotypic frequencies of the C609T polymorphism among cases and controls are presented in Table 1. The genotypic frequencies in both the controls and cases groups were in Hardy–Weinberg equilibrium (*p* = 0.41 and *p* = 0.97, respectively). There were no significant differences in the genotypic and allelic frequencies of the C609T polymorphism between cases and control.

**Table 1:** Risk estimates for *NQO1* variant allele in cases and controls

	Cases *N* (%)	Controls *N* (%)	Univariate OR (95% CI)	Adjusted OR (95% CI)	*p* value
Overall					
CC	190 (48.47)	196 (53.70)	1.00 (ref.)	1.00 (ref.)	
CT	166 (42.35)	147 (40.27)	1.16 (0.86–1.57)	1.18 (0.78–1.62)^a^	0.345
TT	36 (9.18)	22 (6.03)	1.69 (0.96–3.01)	1.61 (0.97–3.05)^a^	0.074
CT + TT	202 (51.48)	169 (46.30)	1.23 (0.93–1.64)	1.34 (0.91–1.65)^a^	0.13
Alleles					
C	546 (69.64)	539 (73.84)	1.00 (ref.)	1.00 (ref.)	
T	238 (30.36)	191 (26.16)	1.23 (0.98–1.54)	1.19 (1.04–1.54)^a^	0.09
Age ≤30					
CC	49 (49.00)	53 (53.54)	1.00 (ref.)	1.00 (ref.)	
CT	45 (45.00)	39 (39.39)	1.28 (0.69–2.23)	1.24 (0.73–2.41)^b^	0.532
TT	6 (6.00)	7 (7.07)	0.93 (0.27–3.06)	0.89 (0.27–3.05)^b^	0.901
CT + TT	51 (51.00)	46 (46.46)	1.20 (0.68–2.10)	1.23 (0.71–2.16)^b^	0.535
Age >30					
CC	141 (48.29)	143 (53.76)	1.00 (ref.)	1.00 (ref.)	
CT	121 (41.44)	108 (40.60)	1.14 (0.80–1.61)	1.10 (0.76–1.60)^b^	0.434
TT	30 (10.27)	15 (5.64)	0.56 (0.28–1.09)	0.61 (0.35–1.12)^b^	0.106
CT + TT	151 (51.71)	123 (46.14)	1.24 (0.89–1.74)	1.36 (0.87–1.70)^b^	0.194
Males					
CC	133 (50.00)	131 (55.27)	1.00 (ref.)	1.00 (ref.)	
CT	109 (40.98)	91 (38.40)	1.18 (0.81–1.71)	1.17 (0.79–1.69)^c^	0.465
TT	24 (9.02)	15 (6.33)	1.57 (0.79–3.19)	1.50 (0.72–3.21)^c^	0.287
CT + TT	133 (50.00)	106 (44.73)	1.23 (0.87–1.75)	1.26 (0.89–1.76)^c^	0.237
Females					
CC	57 (45.24)	65 (50.78)	1.00 (ref.)	1.00 (ref.)	
CT	57 (45.24)	56 (43.75)	1.37 (0.81–2.31)	1.20 (0.76–2.39)^c^	0.193
TT	12 (9.52)	7 (5.47)	1.74 (0.663–4.55)	1.73 (0.66–4.54)^c^	0.299
CT + TT	69 (54.76)	63 (49.22)	1.25 (0.76–2.05)	1.21 (0.73–2.01)	0.376
Never‐smokers					
CC	123 (47.31)	101 (45.09)	1.00 (ref.)	1.00 (ref.)	
CT	115 (44.23)	106 (47.32)	0.89 (0.61–1.29)	0.89 (0.59–1.31)^d^	0.604
TT	22 (8.46)	17 (7.59)	1.06 (0.53–2.13)	1.06 (0.51–2.14)^d^	0.898
CT + TT	137 (52.69)	123 (54.91)	0.91 (0.64–1.31)	0.93 (0.68, 1.35)^d^	0.630
Ever‐smokers					
CC	67 (50.76)	95 (67.38)	1.00 (ref.)	1.00 (ref.)	
CT	51 (38.64)	41 (29.08)	1.76 (1.05–2.96)	1.76 (1.07–2.89)^d^	0.024
TT	14 (10.61)	5 (3.55)	3.94 (1.39–12.73)	3.91 (1.41–13.65)^d^	0.006
CT + TT	65 (49.24)	46 (32.63)	2.00 (1.22, 3.28)	2.05 (1.25–3.32)^d^	0.005

^a^Adjusted by center, age, sex, and smoking status. ^b^Adjusted by center, sex, and smoking status. ^c^Adjusted by center, age, and smoking status. ^c^Adjusted by center, age, and sex.

We further divided the sample according to age or smoking status. Age bimodality is an inherent feature of NPC in North Africa, with a first peak centered around 15 years of age, followed by a slope that increase after 30 and peak in old age (65+) (Bray et al., 2008). As cases from the first age peak (≤30) are believed to have a strongest genetic susceptibility, we tested association between NPC risk and C609T polymorphism in two subgroups, ≤30 and >30 years of age. However, no significant association was observed (Table 1). We also examined the effect of C609T genotypes separately for males and females, and as expected no significant association was observed (Table 1).

When dividing the sample according to smoking status, we observed a strong association between the C609T polymorphism and NPC risk among ever‐smokers. In this group, carriers of the CT and TT genotypes were, respectively, at 1.76 times and 3.91 times higher risk to develop NPC than CC carriers (*p* = 0.024 and *p* = 0.006, respectively; see Table 1). Among nonsmokers, there was no association between the C609T polymorphism and NPC risk. We performed multiple logistic regression to assess the associations between *NQO1* C609T polymorphism and risk of NPC (Table 2). Our results show evidence for gene–environment interaction with an effect of the T allele conditional on smoking status (OR = 1.95, 95% CI = 1.20–3.19; interaction *p* = 0.007). This interaction effect is likely contributing to NPC risk.

**Table 2:** Gene–Environment interaction using logistic regression analysis

Risk factor	*β*	*SE*	Wald *χ* ^2^	*p*‐value	OR^a^	95% CI
NQO1	−0.045	0.145	0.097	0.756	0.956	0.719–1.271
Age	0.000	0.005	0.003	0.959	1.000	0.991–1.010
Sex	−0.351	0.184	3.641	0.056	0.704	0.491–1.010
Smoking	−0.727	0.227	10.226	0.001	0.484	0.310–0.755
*NQO1*∗Smoking	0.672	0.250	7.234	0.007	1.959	1.200–3.197

Interaction between smoking and T allele.

In our North African samples, we found that the *NQO1* C609T polymorphism is significantly associated with risk to UCNT risk among ever‐smokers but not among nonsmokers. There seems to be a significant additive effect between this polymorphism and smoking contributing to onset of NPC.

Among ever‐smokers, the T allele conferred increase of risk in a codominant manner, the OR associated with genotype TT being higher than the one associated with the CT genotype. This codominant effect is compatible with what is known about *NQO1* C609T functionality: It has been reported that subjects with the TT genotype have no detectable NQO1 protein (no enzymatic activity), and those with the CT genotype have intermediate levels of NQO1 protein compared to the CC genotype (Han et al., 2009; Smith, 1999).

A wide variation of the T allele frequency has been observed across ethnic groups. The TT genotype is as rare as 2% in white populations but as frequent as 20% in Asian populations (Kelsey et al., 1997). Our study is the first to document the frequencies of the *NQO1* C609T polymorphism in the North African population. The frequency of the T allele in our population (0.26) was similar to that found in African American population (0.22) (Wiencke, Spitz, McMillan, & Kelsey, 1997), higher than what was reported for example in the Caucasian population (0.14), and lower than what was reported in the Chinese population (0.42) (Zhang, Li, et al., 2003; Zhang, Schulz, et al., 2003).

To date, association of the *NQO1* gene with NPC risk has been investigated in only two studies and results have been inconsistent. In a study of 120 cases and 120 controls from South China, the *NQO1* T allele was significantly more frequent among NPC cases than controls (Wu, 2002), a result consistent with ours. The second study conducted on a larger sample from South China (358 cases and 629 controls) reported no significant differences between cases and controls (Guo et al., 2009). These two studies were conducted in populations of high NPC incidence (endemic area). In none of the studies, authors subdivided by smoking status in their analysis, this makes the comparison with the current study difficult.

Several studies have examined the relationship between the *NQO1* genetic polymorphisms and tobacco‐related cancers, including lung, bladder, colorectal (Chao, Zhang, Berthiller, Boffetta, & Hashibe, 2006), esophageal (Zhang, Li, et al., 2003; Zhang, Schulz, et al., 2003), and head and neck (Cho et al., 2006), but the findings have been relatively inconsistent and varies across ethnic groups. A high‐quality meta‐analysis of the *NQO1* C609T polymorphism and risk of bladder, lung, and colorectal cancer revealed that there was significant heterogeneity across ethnic groups: The T allele was associated with an increased risk of bladder and colorectal cancer among whites, whereas a protective role was observed for the T allele among Asian populations (Chao et al., 2006). Regarding lung cancer, the deleterious effect of the T allele among whites did not reach significance (due to low statistical power) but its protective effect among Asian populations was significant. For bladder cancer in white populations, the increase of risk was consistent with a codominant effect and further stratification by smoking status suggested a possible modification by ever smoking (Chao et al., 2006). Such results are very similar to the ones we report here for NPC, the North African population being considered as Caucasian and being genetically much nearer to the European/American white populations than to Asian populations.

A more recent meta‐analysis of 23 studies focusing on lung cancer confirmed that the T allele was associated with a slight increase of risk among whites but not among Asians (Liu & Zhang, 2011). This meta‐analysis was not taking smoking status into account. Three individual studies did so, two of them reported weak associations between the C allele and risk of lung cancer in Asian populations, modulated, or not, by smoking (Chen, Lum, Seifried, Wilkens, & Marchand, 1999; Lin et al., 2003). The last study conducted in United Kingdom on 84 cases and 145 controls reports a significant association between the T allele and non‐small‐cell lung cancer in heavy smokers (Lewis, Cherry, Niven, Barber, & Povey, 2001). Such a result is comparable to the one we obtained with NPC in the North African population.

The fact that associations between the *NQO1* C609T polymorphism and cancers vary between Caucasian/whites and Asian populations is well discussed in the paper of Chao et al. In brief, the difference could be due to (a) an interaction between the dual function of the NQO1 enzyme and the environmental exposure which varies between Asia and the West (e.g., smoking of low‐tar cigarettes); (b) other genetic mechanisms present in the Asian population that compensate more effectively for the loss of the detoxifying activity associated with the T allele, and (c) difference in linkage disequilibrium across ethnic groups: other functional variants in linkage disequilibrium with the C609T polymorphism may be implicated, notably in Asian populations (Chao et al., 2006).

To the best of our knowledge, this is the first study investigating a possible gene–environment interaction between smoking and the *NQO1* C609T polymorphism on NPC. A significant interaction was found between the *NQO1* 609 T allele and smoking status, thus providing evidence that this genetic polymorphism can modify the effect of smoking on NPC risk, although the mechanism through which smoking and the *NQO1* 609 T variant interact to affect NPC is unknown. Molecular studies pointed out that cigarette smoke is a factor for tumor growth that may act as a mutagen and DNA damaging agent that drives tumor initiation in normal epithelial cells of the nasopharynx (Furmanski, 2013; Salem et al., 2013). The two‐electron reduction activity catalyzed by NQO1 is of benefit to the cell as it prevents generation of free radicals through redox cycling; thus, NQO1 protects the cells from oxidative stresses and prevents carcinogenesis (Atia, Alrawaiq, & Abdullah, 2014; Cuendet, Oteham, Moon, & Pezzuto, 2006; Nebert, Roe, Vandale, Bingham, & Oakley, 2002). Therefore, it is biologically plausible that those harboring the *NQO1* 609 CT or TT genotypes, which are expected to have lower enzyme activity, may have a higher NPC risk when exposed to cigarette smoke. However, direct evidence for the biological significance of the interaction between *NQO1* C609CT polymorphism and smoking awaits further investigations.

## 4 CONCLUSION

To conclude, our data provide evidence of an association of the *NQO1* C609T polymorphism with the risk of NPC among smokers in North Africa. This association is consistent with association observed in Caucasian populations between the C609T polymorphism and other cancers. In addition, the interactions between *NQO1* C609T polymorphism and smoking status appear to increase risk to NPC. However, further studies are needed to confirm these results.

## CONFLICT OF INTEREST

The authors declare that they have no competing interests.
